# Accuracy of Intraocular Lens Power Calculation Formulas for Highly Myopic Eyes

**DOI:** 10.1155/2016/1917268

**Published:** 2016-03-29

**Authors:** Yichi Zhang, Xiao Ying Liang, Shu Liu, Jacky W. Y. Lee, Srinivasan Bhaskar, Dennis S. C. Lam

**Affiliations:** ^1^State Key Laboratory of Ophthalmology, Zhongshan Ophthalmic Center, Sun Yat-sen University, Guangzhou 510080, China; ^2^Dennis Lam & Partners Eye Center, Suite 1515, Central Building, 1-3 Peddler Street, Central, Hong Kong; ^3^C-MER (Shenzhen) Dennis Lam Eye Hospital, Shenzhen 518000, China

## Abstract

*Purpose.* To evaluate and compare the accuracy of different intraocular lens (IOL) power calculation formulas for eyes with an axial length (AL) greater than 26.00 mm.* Methods.* This study reviewed 407 eyes of 219 patients with AL longer than 26.0 mm. The refractive prediction errors of IOL power calculation formulas (SRK/T, Haigis, Holladay, Hoffer Q, and Barrett Universal II) using User Group for Laser Interference Biometry (ULIB) constants were evaluated and compared.* Results.* One hundred seventy-one eyes were enrolled. The Barrett Universal II formula had the lowest mean absolute error (MAE) and SRK/T and Haigis had similar MAE, and the statistical highest MAE were seen with the Holladay and Hoffer Q formulas. The interquartile range of the Barrett Universal II formula was also the lowest among all the formulas. The Barrett Universal II formulas yielded the highest percentage of eyes within ±1.0 D and ±0.5 D of the target refraction in this study (97.24% and 79.56%, resp.).* Conclusions.* Barrett Universal II formula produced the lowest predictive error and the least variable predictive error compared with the SRK/T, Haigis, Holladay, and Hoffer Q formulas. For high myopic eyes, the Barrett Universal II formula may be a more suitable choice.

## 1. Introduction

High myopia or pathological myopia is associated with elongation of the axial length (AL) longer than 26 mm or a refractive error of at least −6 diopters (D). High myopia is one of the most prevalent refractive conditions globally with a higher risk of other eye conditions [1–7]. The prevalence of high myopia has been estimated in several large-scale population studies. In the Beijing Eye Study [4], the prevalence of high myopia was 0.98%: 0.53% in central India [3], 2.7% in Europe [8], and 8.4% of adults aged over 40 years in Singapore [5]. Moreover, in high myopic eyes, the incidence of cataract is significantly higher than in nonmyopic eyes, and the progression is also faster [9], possibly due to the proinflammatory internal microenvironment in the high myopic eye [10].

Calculation of intraocular lens power (IOL) in high myopic eyes remains a challenge, often leading to unexpected postoperative hyperopia [11–15]. The main potential sources of error in IOL calculation for high myopic eyes include AL measurement, IOL constants used, and IOL power calculation formula employed. In high myopic eyes, due to the presence of posterior staphyloma, partial coherence interferometry (PCI) may be better than conventional ultrasound for measuring the AL [16, 17]. Furthermore, the prediction of refractive accuracy may be improved by adjusting the AL by formulas derived from regression analysis [15]. In terms of the IOL constants used in IOL power calculation formulas, it has been reported that optimized constants greatly improve the predictive refraction outcomes [12, 13, 18]. Currently, the constants of User Group for Laser Interference Biometry (ULIB) are widely used for high myopic eyes. Studies have suggested that the ULIB constants are more accurate than manufacturer-recommended IOL constants for long eyes [11, 13]. The most studied IOL power calculation formulas include third-generation formulas (Holladay 1, SRK/T, and Hoffer Q) and fourth-generation formulas (Haigis and Holladay 2) [12–14, 19–23]. Recently, a new generation formula Barrett Universal II has become available for commercial use and its performance showed promise in 1 previous study [13].

In the present study, the accuracy of IOL power calculation formulas (SRK/T, Haigis, Hoffer Q, Holladay 1, and Barrett Universal II) using ULIB constants was evaluated and compared in eyes with AL greater than 26.0 mm.

## 2. Patients and Methods

### 2.1. Patients

Local ethical approval was obtained from the ethics committee of C-MER (Shenzhen) Dennis Lam Eye Hospital for this retrospective study. The medical charts of consecutive cataract surgery patients with AL longer than 26.0 mm in the operated eye(s) were reviewed. Cataract extraction with IOL implantation was performed at C-MER (Shenzhen) Dennis Lam Eye Hospital (Shenzhen, China) or Dennis Lam & Partners Eye Center (Hong Kong, China) by the same surgeon (Dr. Dennis S. C. Lam). Cases from January 1, 2013, to June 30, 2015, were reviewed.

One eye was randomly selected from each patient if both eyes had AL greater than 26.00 mm. For subjects with only one eye with AL greater than 26.00 mm, that eye was selected for analysis. The inclusion criteria were as follows: (1) biometric measurements determined by PCI (IOLMaster, Software V5.4 and above, Carl Zeiss Meditec, Inc., Dublin, CA, USA); (2) cataract surgery performed by phacoemulsification and in-the-bag IOL implantation; (3) use of the AcrySof IOL; and (4) 2.75 mm clear corneal incisions located temporally or superiorly. The exclusion criteria were as follows: (1) patients with a history of previous intraocular surgery or intraoperative or postoperative complications; (2) preexisting ocular diseases that may influence postoperative refraction, including keratoconus, corneal scarring, endothelial dystrophy, retinal detachment, and macular edema; (3) patients who underwent combined surgical procedures; (4) patients with follow-up of less than 1 month.

The patients' AL, anterior chamber depth (ACD), and keratometric (*K*) (both *K*_1_ and *K*_2_) values were collected for the backcalculation of formulas.

### 2.2. Formulas and Lens Constants

The SRK/T, Haigis, Holladay 1, and Hoffer formulas were calculated or backcalculated, using the ULIB constants in the IOLMaster. The backcalculation with the new generation Barrett Universal II formula was performed using the online software (http://www.apacrs.org/barrett_universal2/); the constants recommended in this online software were used for the backcalculation.

### 2.3. Evaluation of the Accuracy in Predicted Refraction

In most cases, the target refraction was low myopia (≤3.0 D). The postoperative actual refraction values were obtained at least 1 month after surgery. The refractive prediction error was calculated as the difference between the actual postoperative refractive outcome and the predicted refraction (actual postoperative refraction − predicted refraction) for each formula. The mean numerical error (MNE), mean absolute error (MAE), and median absolute error were calculated for each formula. The percentage of eyes that were within ±0.5 D and ±1.0 D of the target refraction was calculated for each formula. To further clarify the relationship between AL and predicted refraction error, the association analysis between refraction prediction error and AL was performed for each formula.

### 2.4. Statistical Analysis

The differences in the MNE, MAE, median absolute error, and the percentages of eyes within ±0.5 D and ±1.0 D of the target refraction between formulas were assessed using the Wilcoxon signed-rank test or Chi-Square test. The Bonferroni correction was used for multiple comparisons. The association between refraction prediction error and AL was assessed using Spearman's rank correlation. *P* values less than 0.05 were considered statistically significant. Statistical analyses were performed using SPSS software (version 19.0, SPSS, Inc., Chicago, IL, USA) and Microsoft Excel (Microsoft Corporation, Redmond, Washington, USA). Means were expressed as mean ± standard deviation (SD).

## 3. Results

The records of 407 eyes of 219 high myopic patients who underwent phacoemulsification and IOL implantation were reviewed. Finally, 171 eyes of 171 subjects were included in this study.  Table 1 summarizes the demographics and preoperative biometrics of patients included in this study.  Table 2 shows the implanted IOL model and the frequency of their implantation. All 12 eyes with minus IOL power and the single eye with zero-diopter IOL were implanted with the AcrySof MA60MA.  Table 3 summarizes the MNE and MAE for formulas.

### 3.1. Numerical Predictive Error

**Figure 1:**
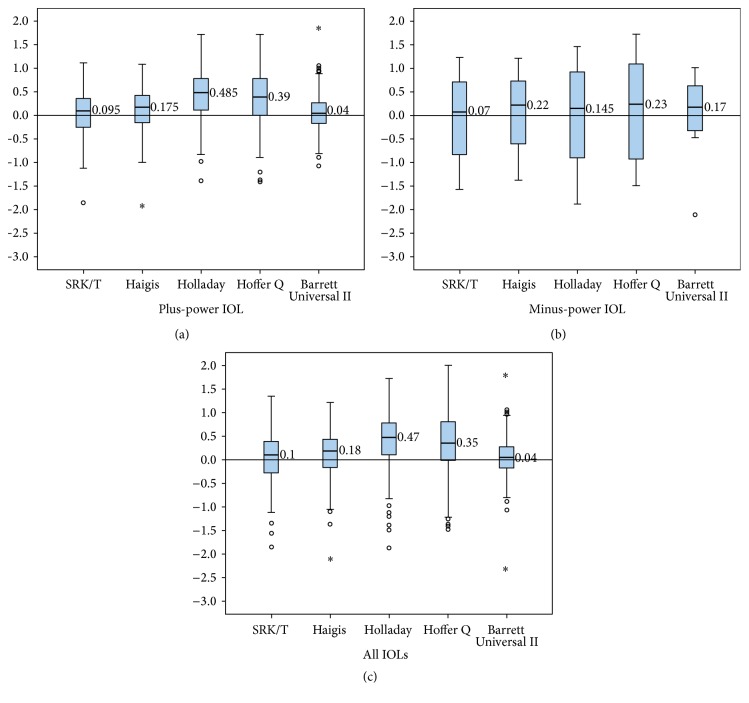
Mean numerical error in Group A and Group B. Group A were eyes with plus-power IOL; Group B were eyes with minus-power IOL. *x* axis was formulas; *y* axis was mean numerical error.

In eyes with plus-power IOL, all formulas had a positive MNE and median numerical error (Table 3 and Figure 1). In eyes with minus-power IOL, all formulas had a positive median numerical error (Figure 1). Furthermore, all formulas yielded a considerably higher interquartile range (IQR, as a measure of statistical dispersion, being equal to the difference between the upper and lower quartiles) and SD compared to eyes with plus-power IOL. The IQR of the Barrett Universal II formula was smaller than that of the other formulas in both eyes with plus-power IOL and minus-power IOL (Figure 1 and Table 4).

In all eyes, the MNE values ranged from 0.03 to 0.40 (Table 3). All formulas had a positive MNE and median numerical error (Table 3 and Figure 1). The IQR of the Barrett Universal II formula was also the smallest among all formulas (Figure 1 and Table 4).

### 3.2. Absolute Predictive Error

In eyes with plus-power IOL, the MAE values ranged from 0.31 to 0.59 (Table 3) and with minus-power IOL, the MAE values ranged from 0.56 to 0.9 (Table 3). In all eyes, the MAE values ranged from 0.33 to 0.62 (Table 3); the Barrett Universal II formula yielded the lowest MAE among these formulas and Haigis and SRK/T formulas had similar MAE, but they had MAE lower than that of the Holladay and Hoffer Q formulas (Table 5).

**Figure 2:**
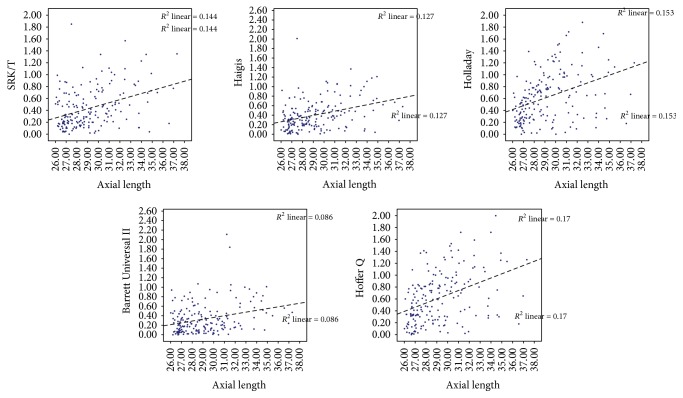
The association between absolute prediction error and axial length. *x* axis was axial length; *y* axis was absolute prediction error of each formula. The refractive errors were higher as the axial length became longer for all formulas.

To further investigate the association between refraction prediction error and AL, we performed correlation analysis between absolute predictive error and AL for all formulas. The refraction prediction error of all formulas had a positive association with AL (Figure 2 and Table 6).

### 3.3. Eyes within ±0.50 D and ±1.00 D of the Target Refraction

**Figure 3:**
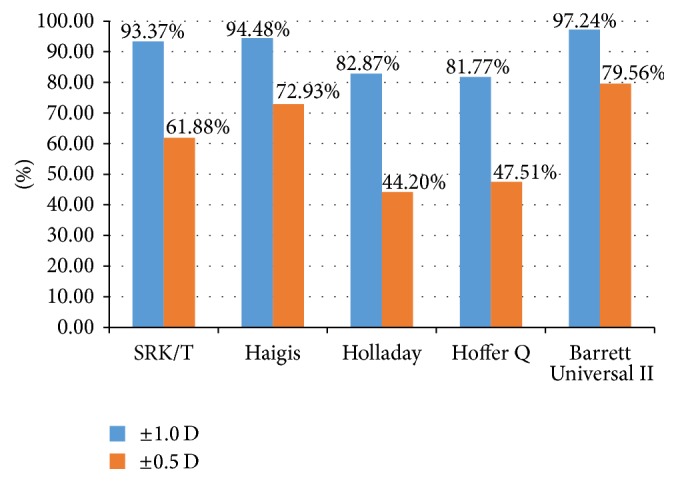
Percentages of eyes within both ±0.5 D and ±1.0 D of the target refraction.

In our study, the SRK/T, Haigis, and Barrett Universal II formulas yielded similar percentages of eyes within ±1.00 D of the target refraction, while the Holladay and Hoffer Q formulas gave lower percentages (Table 7 and Figure 3) (*P* < 0.005). The Haigis and Barrett Universal II formulas yielded similar percentages of eyes within ±0.50 D of the target refraction, while the SRK/T (compared with Barrett Universal II), Holladay, and Hoffer Q formulas gave lower percentages (Table 7 and Figure 3) (*P* < 0.005). The Barrett Universal II formula yielded the highest percentages of eyes within both ±0.50 D and ±1.00 D of the target refraction, although there was no statistical difference with the Haigis formula (Table 7 and Figure 3).

## 4. Discussion

In the present study, we compared the accuracy of four widely used IOL power calculation formulas, namely, Holladay 1, SRK/T, Hoffer Q, and Haigis, and a new generation formula, the Barrett Universal II, for 171 high myopic eyes with AL greater than 26.00 mm. To the best of our knowledge, this is one of the largest studies to date investigating the accuracy of IOL calculations comparing five IOL formulas for high myopic eyes. This group of patients deserve special attention as they are predisposed to biometrical measurement inaccuracies leading to refractive surprises and we have a high prevalence of myopia in our locality. Furthermore, this is also one of the few studies, supporting the use of the newer Barrett Universal II formula to enhance predictability in high myopic eyes.

In recent years, two main challenges in IOL power calculation for high myopic eyes have been encountered: (1) there are always unexpected hyperopic outcomes with IOL power calculation formulas, regardless of whether the eyes are plus-power IOL or minus-power IOL, although this tendency for postoperative hyperopic outcomes is more marked for eyes with minus-power IOL; (2) as the AL increases, the refractive prediction errors of formulas also escalate, particularly in the eyes with minus-power IOL. To avoid these postoperative hyperopic outcomes and achieve higher patient satisfaction, surgeons usually empirically set a target refraction of −1.00 D to −3.00 D.

The most important reasons for inaccuracy in IOL calculations are errors in measuring AL, the effective lens position (ELP) location assumption, and the IOL constant used. Measurement of AL using partial coherence interferometry is more accurate than conventional ultrasound; however, since it assumes a standard value for the refractive index of the eye, it may be a source of error in highly myopic eyes where the vitreous is more liquefied [15]. To account for the potential errors from AL measurement, Wang and his team described an AL-adjusted formula, based on regression analysis [15]. Nevertheless, studies have shown that the AL-adjusted method overcompensates for IOL power of more than 6.00 D, although it might be more accurate in patients requiring an IOL of less than 6.00 D. It is, however, an empirically derived formula and most surgeons do not adjust the AL.

The IOL constants can be another source of error. Haigis explained the reasons for increased error in eyes with longer AL using model calculations and showed how the geometry of the IOL changed the principal optical plane of the lens. Not accounting for this could lead to erroneous IOL calculation, whereas adjusting for this leads to better outcomes in myopic eyes [9, 10, 16, 24]. Abulafia et al. also suggested a need for different constants for plus- and minus-power lenses [13]. At present, optimized IOL constants from the ULIB are widely used in IOL power calculation, and studies have suggested that the ULIB constants are more accurate than manufacturer-recommended IOL constants for calculations in highly myopic eyes [12, 13]. In this study, we used ULIB constants and achieved considerable lower refractive prediction error with the Haigis and SRK/T formulas for eyes with plus-power IOL.

Another source of error is the ELP. The new Barrett Universal II formula uses a lens factor that considers both the physical position and the location of the principal planes of the IOL, although the details of this formula are still unknown [13, 25].

An unexpected hyperopic outcome was found with all formulas, and the predicted error had a tendency to be greater in eyes with minus-power IOL. In our study, the Barrett Universal II formula had the lowest predicted error and SRK/T and Haigis formulas had a similar accuracy and lower refractive predicted error than the Holladay and Hoffer Q formulas. Furthermore, the IQR of the Barrett Universal II formula was the lowest of all the formulas; this together with the MAE results and the fact that it produced the maximum number of eyes within ±0.50 and 1.00 D indicate that the Barrett Universal II formula produced the least variable predictive error. This suggests that this formula may be better for calculating IOL power in high myopic eyes.

A limitation of this study was that the number of eyes with minus-power IOL and zero-diopter IOL was small; this may have been insufficient in assessing the performance of the various formulas in eyes with minus-power IOL and zero-diopter IOL. Although in real life scenarios, like in our study, only a relatively small number of patients in the general population will need to use the minus-power IOL, further studies involving more eyes requiring a minus-power IOL and zero-diopter IOL may be warranted.

## 5. Conclusions

In conclusion, the results of this study suggested that, for high myopic eyes, the Barrett Universal II formula provides the most predictable outcomes. The SRK/T and Haigis formulas, employing ULIB constants, performed similarly but better than the Holladay and Hoffer Q formulas. Further studies involving eyes with minus-power IOL and zero-diopter IOL are warranted to further assess the accuracy of this formula for these subgroups.

## Figures and Tables

**Table 1:** Demographics and preoperative biometrics of the study population.

Parameter	Value			
	Plus-power IOL	Minus-power IOL	Zero-diopter IOL	All
Enrolled subjects (*n*)	158	12	1	171
Eyes (*n*)	158	12	1	171
Gender, *n* (%):				
Male	80 (51%)	6 (50%)	1	87 (51%)
Female	78 (49%)	6 (50%)	—	84 (49%)
Age (y)				
Mean ± SD	57.72 ± 12.61	51 ± 15	41	57.65 ± 12.53
Range	20 to 92	32 to 80	—	20 to 92
Axial length (mm)				
Mean ± SD	28.77 ± 2.15	33.81 ± 1.90	34.44	29.14 ± 2.50
Range	26.01 to 34.63	30.20 to 37.31	—	26.01 to 37.31
Anterior chamber depth (mm)				
Mean ± SD	3.47 ± 0.39	3.34 ± 0.47	3.45	3.46 ± 0.40
Range	2.34 to 4.17	2.57 to 5.11	−	2.34 to 5.11
Keratometric value (D)				
Mean ± SD	43.51 ± 2.19	44.62 ± 2.24	43.53	43.61 ± 2.22
Range	40.36 to 48.05	41.95 to 48.19	—	40.36 to 48.19
IOL power (D)				
Mean ± SD	9.16 ± 4.24	−2.75 ± 1.42	0	8.21 ± 5.28
Range	+1.00 to +20.00	−5.00 to −1.00	—	−5.00 to +20.0

**Table 2:** Brand and model of the implanted IOLs.

IOL model	Eyes (*n*)	Percentage (%)
AcrySof MA60MA	12	8%
AcrySof SA60AT	109	64%
AcrySof SN60WF	16	9%
AcrySof SN60TA	14	8%
AcrySof SN60AD1/3	2	1%

**Table 3:** Mean numerical error (MNE) and mean absolute error (MAE) for formulas and groups.

Formula	Plus-power IOL		Minus-power IOL		Zero-power IOL		All	
	MNE (D)	MAE (D)	MNE (D)	MAE (D)	MNE (D)	MAE (D)	MNE (D)	MAE (D)
SRK/T								
Mean ± SD	0.03 ± 0.50	0.39 ± 0.30	−0.06 ± 0.92	0.78 ± 0.46	1.34	1.34	0.03 ± 0.55	0.43 ± 0.34
Range	−1.85 to 1.11	0.00 to 1.85	−1.57 to 1.23	0.04 to 1.57	—	—	−1.85 to 1.34	0.00 to 1.85
Haigis								
Mean ± SD	0.13 ± 0.44	0.37 ± 0.28	0.08 ± 0.83	0.71 ± 0.50	1.18	1.18	0.13 ± 0.49	0.40 ± 0.31
Range	−2.01 to 1.08	0.00 to 2.01	−1.27 to 1.21	0.04 to 1.37	—	—	−2.01 to 1.21	0.00 to 2.01
Holladay								
Mean ± SD	0.43 ± 0.55	0.59 ± 0.38	−0.04 ± 1.04	0.87 ± 0.53	1.69	1.69	0.40 ± 0.63	0.62 ± 0.41
Range	−1.39 to 1.72	0.00 to 1.72	−1.20 to 3.34	0.11 to 1.88	—	—	−1.88 to 1.72	0.00 to 1.88
Hoffer Q								
Mean ± SD	0.37 ± 0.59	0.57 ± 0.39	0.05 ± 1.05	0.9 ± 0.13	2	2	0.35 ± 0.66	0.61 ± 0.43
Range	−1.41 to 1.72	0.00 to 1.72	−1.49 to 1.72	0.18 to 1.72	—	—	−1.49 to 2.00	0.00 to 2.00
Barrett Universal II								
Mean ± SD	0.04 ± 0.42	0.31 ± 0.29	0.08 ± 0.76	0.56 ± 0.49	0.71	0.71	0.05 ± 0.46	0.33 ± 0.32
Range	−1.07 to 1.84	0.00 to 1.84	−2.11 to 1.01	0.10 to 2.11	—	—	−2.11 to 1.84	0.00 to 2.11

**Table 4:** Percentile rank of the numerical predicted error.

Formula	All				Plus-power IOL				Minus-power IOL			
	25%	50%	75%	IQR	25%	50%	75%	IQR	25%	50%	75%	IQR
SRK/T	−0.28	0.10	0.39	0.67	−0.26	0.10	0.36	0.62	−0.87	0.70	0.72	1.59
Haigis	−0.18	0.18	0.45	0.62	−0.16	0.18	0.43	0.59	−0.61	0.22	0.74	1.35
Holladay	0.10	0.47	0.79	0.69	0.11	0.49	0.78	0.67	−1.02	0.15	0.94	1.96
Hoffer Q	0.02	0.35	0.81	0.83	0.00	0.39	0.78	0.78	−1.03	0.23	1.11	2.13
Barrett Universal II	−0.18	0.04	0.28	0.46	−0.17	0.04	0.27	0.44	−0.36	0.17	0.66	1.02

**Table 5:** Difference in absolute error between different formulas.

Paired group	*P* values
Haigis-SRK/T	0.062
Holladay-SRK/T	0.000^*∗*^
Hoffer Q-SRK/T	0.000^*∗*^
Holladay-Haigis	0.000^*∗*^
Hoffer Q-Holladay	0.740
Hoffer Q-Haigis	0.000^*∗*^
Barrett Universal II-SRK/T	0.000^*∗*^
Barrett Universal II-Haigis	0.000^*∗*^
Barrett Universal II-Holladay	0.000^*∗*^
Barrett Universal II-Hoffer Q	0.000^*∗*^

^*∗*^Statistical significance; *α* = 0.005.

**Table 6:** Association between absolute predicted error and axial length.

Formula	Correlation coefficient	*P* value
SRK/T	0.360	0.000
Haigis	0.367	0.000
Holladay	0.445	0.000
Hoffer Q	0.428	0.000
Barrett Universal II	0.259	0.000

**Table 7:** Difference in eyes within ±0.5 D and ±1.0 D of the target refraction between different formulas.

Paired group	*P* values	
	±1.0 D	±0.5 D
Haigis-SRK/T	0.660	0.025
Holladay-SRK/T	0.002^*∗*^	0.001^*∗*^
Hoffer Q-SRK/T	0.001^*∗*^	0.006
Holladay-Haigis	0.000^*∗*^	0.000^*∗*^
Hoffer Q-Holladay	0.000^*∗*^	0.000^*∗*^
Hoffer Q-Haigis	0.000^*∗*^	0.000^*∗*^
Barrett Universal II-SRK/T	0.082	0.000^*∗*^
Barrett Universal II-Haigis	0.187	0.138
Barrett Universal II-Holladay	0.000^*∗*^	0.000^*∗*^
Barrett Universal II-Hoffer Q	0.000^*∗*^	0.000^*∗*^

^*∗*^Statistical significance; *α* = 0.005.
